# Prognostic Evaluation of Lower Third Molar Eruption Status from Panoramic Radiographs Using Artificial Intelligence-Supported Machine and Deep Learning Models

**DOI:** 10.3390/bioengineering12111176

**Published:** 2025-10-29

**Authors:** Ipek N. Guldiken, Alperen Tekin, Tunahan Kanbak, Emine N. Kahraman, Mutlu Özcan

**Affiliations:** 1 Department of Oral and Maxillofacial Surgery, Faculty of Dentistry, Istinye University, Ayazağa Mahallesi, Azerbaycan Caddesi, Vadistanbul 4A Blok, 34396 Sariyer, Istanbul, Turkey; 2 Department of Oral and Maxillofacial Radiology, Faculty of Dentistry, Istanbul Medeniyet University, Fatih Mahallesi, Eski Ankara Asfaltı Caddesi No:28, 34956 Tuzla, Istanbul, Turkey; alperentekin37@gmail.com; 3 Independent Researcher, 8032 Copenhagen, Denmark; kanbaktunahan@gmail.com; 4 Faculty of Dentistry Department of Oral and Maxillofacial Radiology, Istinye University, Ayazağa, Defne Street No:1 Apartment:34408, 34408 Sariyer, Istanbul, Turkey; eminenur.kahraman@istinye.edu.tr; 5 Clinic of Masticatory Disorders and Dental Biomaterials, Center for Dental Medicine, University of Zurich, Plattenstrasse 11, 8032 Zurich, Switzerland; mutluozcan@hotmail.com

**Keywords:** artificial intelligence, deep learning, panoramic radiographs, prognostic assessment, third molar eruption

## Abstract

The prophylactic extraction of third molars is highly dependent on the surgeon’s experience as the common practices and guidelines contradict. The purpose of this study was to evaluate the eruption status of impacted third molars using deep learning-based artificial intelligence (AI) and to develop a model that predicts their final positions at an early stage to aid clinical decisions. In this retrospective study, 1102 panoramic radiographs (PANs) were annotated by three expert dentists to classify eruption status as either initial or definitive. A dataset was created and two deep learning architectures, InceptionV3 and ResNet50, were tested through a three-phase protocol: hyperparameter tuning, model evaluation, and assessment of preprocessing effects. Accuracy, recall, precision, and F1 score were used as performance metrics. Classical machine learning (ML) algorithms (SVM, KNN, and logistic regression) were also applied to features extracted from the deep models. ResNet50 with preprocessing achieved the best performance (F1 score: 0.829). Models performed better with definitive cases than with initial ones, where performance dropped (F1 score: 0.705). Clinically, the model predicted full eruption or impaction with 83% and 75% accuracy, respectively, but showed lower accuracy for partial impactions. These results suggest that AI can support early prediction of third molar eruption status and enhance clinical decision-making. Deep learning models (particularly ResNet50) demonstrated promising results in predicting third molar eruption outcomes. With larger datasets and improved optimization, AI tools may achieve greater accuracy and support routine clinical applications.

## 1. Introduction

The eruption of the third molars, also known as wisdom teeth, usually occurs uneventfully between the ages of 18 and 24. However, in some cases, they may remain impacted due to misalignment or obstruction by other teeth or the periodontium. Historically, prophylactic extraction of these teeth has been recommended to prevent potential complications in the future. However, guidelines published by the National Institute for Health and Care Excellence (NICE) in 2000 state that prophylactic extraction is not necessary if these teeth are not symptomatic [1,2]. Conversely, tooth follicles have been shown to play a critical role in odontogenesis, gingival formation and tooth eruption. However, if not erupted, these follicles have the potential to become permanently embedded in the jawbone and undergo pathological changes with age [3]. Therefore, the prophylactic removal of dental follicles, especially those that may present complications during eruption or fail to erupt completely, is a common practice of paramount importance to general oral health and adjacent teeth. This is particularly important in the case of lower third molars [1,3]. The primary consideration for the clinician in determining the need for prophylactic extraction is the anticipated change in the eruption position of the teeth and the potential implications for future complications.

The current decision-making process for third molar extraction is based on a comprehensive set of risk factors, as outlined in national and international protocols. These factors include anatomical structures, general health, age, dental status, medication history and other patient-, surgeon- and cost-related considerations. The decision to extract the third molar is then made on the basis of a comprehensive prognostic evaluation of the clinical data in conjunction with radiological information obtained from preoperative panoramic radiographs, PAN. The most commonly considered criteria for this decision are the potential for healthy eruption of the third molar and its relationship to neighboring teeth [4].

**Figure 1:**
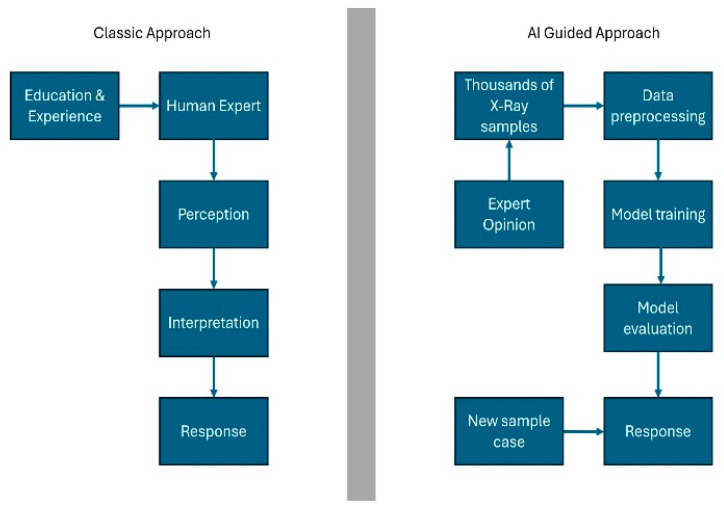
Natural intelligence and artificial intelligence. Quoted and modified from Schwendicke et al. (2020) [5].

Natural intelligence is shaped by perception, interpretation, and biological reactions. While the capacity for computer intelligence to fully replicate human reactions remains limited, it provides substantial support to human interpretation and action processes. In the context of classical machine learning, relevant features are identified and imparted to the model by human experts. In contrast, in deep learning, a sophisticated form of machine learning, these features are learnt in a single step without the need for human intervention. This capacity to process complex data structures, such as images and language, is a significant advancement in the field [5] (Figure 1).

The term “artificial intelligence” (AI) is defined as the ability of machines to imitate human intelligence in order to perform complex tasks. AI has been successfully implemented in numerous areas of daily life, including search engines, online assistants, and gaming. It is increasingly being developed in various fields, including medicine. Radiology, in particular, is regarded as a more accessible area for the integration of AI in medicine, given its utilization of digitally encoded images [6]. Machine learning (ML) constitutes a significant sub-branch of AI. It enables computer models to acquire knowledge and formulate predictions by identifying patterns [7] (Figure 1). In the disciplines of dentistry and maxillofacial radiology, AI has demonstrated considerable potential in a number of domains. These include the localization of root canal orifices, the detection of vertical root fractures, and the identification of dental caries [6,8]. Nevertheless, the focus of the present study was the under-evaluation of clinical applications in this field. While the utilization of AI in dentistry holds promise in the diagnosis of various oral diseases, its integration into routine practice remains limited [7]. The primary issues that must be resolved before the application of AI in dentistry can be realized on a wider scale are as follows: the size of the datasets, the lack of reporting standards, and the need for further research before use in clinical routine [8].

In previous research, AI-based machine and deep learning methods have demonstrated favorable outcomes in the determination of tooth positions on panoramic radiographs and in the diagnosis of various dental pathologies. For instance, Choi et al. sought to ascertain the positional relationship between the mandibular third molar (M3) teeth and the inferior alveolar nerve (IAS) through the implementation of AI, demonstrating that AI exhibited superior accuracy in comparison to maxillofacial surgery specialists, particularly in the context of buccal–lingual position determination [9]. Furthermore, Orhan et al. emphasized that utilizing CBCT data allows for the determination of the relationship between impacted third molars and surrounding anatomical structures through artificial intelligence (AI), in a manner analogous to manual examinations [10]. Furthermore, Bui et al. developed an AI model to determine the index of third molar maturity (I3M) in the field of forensic dentistry, thereby demonstrating the capacity of AI to support clinical decision-making processes [11]. Although these studies have drawn attention to the potential of AI to overcome diagnostic challenges in dentistry, they have generally focused on issues such as position determination, anatomical structure detection, or maturity assessment. However, the primary aim of the present study was to achieve a prognostic ability of whether the teeth will erupt in their natural position, with a view to enhancing the predictive power, particularly with regard to the detection of impacted teeth. In addition to the future positioning of impacted teeth, this can be a precursor for early diagnosis of conditions such as caries or pericoronitis, which are the main factors for prophylactic extraction of these teeth.

The present model has been formulated to pave the way for a future, large-scale dataset that will support the creation of a deep learning model that can diagnose the presence of pathologies requiring the extraction of the relevant tooth on panoramic radiographs at an early stage. The study’s unique contribution lies in its integration of artificial intelligence (AI) into the existing research framework. Unlike previous AI research, this study utilizes AI to predict the eruption of fully or partially impacted teeth, thereby providing clinicians with valuable insights into the clinical decision-making process.

## 2. Materials and Methods

### 2.1. Ethical Approval, Consent and Guiding Standards

Ethical approval for the planned retrospective study was obtained from the Istinye University Ethics Committee (meeting no: 2024/03; protocol no: 24-58). In order to use the data for the study, consent was obtained through the informed consent section, which is routinely included in the standard informed consent form given to patients prior to undergoing imaging procedures. These data (patients’ panoramic radiographs) were thoroughly anonymized and the ethical standards of the Declaration of Helsinki were followed [12]. The development and evaluation of the artificial intelligence/deep learning model used in the study followed the TRIPOD (Transparent Reporting of a multivariable prediction model for Individual Prognosis Or Diagnosis) guidelines [13].

### 2.2. Dataset and Study Design

#### 2.2.1. Radiographic Image Acquisition

The radiographic images used in this study are classified as panoramic dental radiographs. Multiple PAN images of the same patients were obtained from the dental radiography database of Istanbul Medipol University Faculty of Dentistry, Esenler Hospital. In order to ensure standardization, all radiographs were acquired with the same digital panoramic unit (VistaPano S; Dürr Dental AG, Bietigheim-Bissingen, Germany) operated at 73 kVp and 10 mA, with a 13.5 s exposure. Under the supervision of the same radiology technician, images were exported in 16-bit grayscale with a fixed matrix size of 2880 × 1504 pixels and archived in the database.

The PAN database, initiated in 2015, was thoroughly reviewed and patients who met the following inclusion criteria were selected:

- At least two PAN images taken at different time points,

- A minimum interval of one year between consecutive images,

- The presence of a mandibular third molar.

For the selected images, a region of interest (ROI) was defined to include the right and/or left mandibular third molars and surrounding structures, specifically the lower border of the mandible, the external border of the ramus, the occlusal level of the semi-sided molars, and the mesial portion of the adjacent molar. This ROI was used for evaluation and annotation by three dental experts: an oral and maxillofacial surgeon (IG), an oral and maxillofacial radiologist (AT), and an orthodontist (EB).

Initially, cropped sections of each patient’s most recent radiograph were tagged. The aim of the labeling process was to classify the anatomical position of the mandibular third molar in the most recent radiograph available where the tooth had erupted beyond its expected eruption period. Classification categories regarding eruption level included impacted, partially impacted, and erupted. In addition to this primary classification, each image slice was further assigned a subclassification label based on the certainty of the tooth’s final position. The latest PAN of each patient was labeled as definitive, indicating that the tooth’s position was definitive, while all earlier images were labeled as initial, reflecting the possibility of positional change (i.e., the tooth could remain impacted, fully erupt, or exhibit a partially impacted position). The primary objective of using multiple radiographic images for a single patient is to evaluate the ability of the AI model to predict future outcomes based on the final image as a reference. This analysis constitutes the core aim of the study.

**Figure 2:**
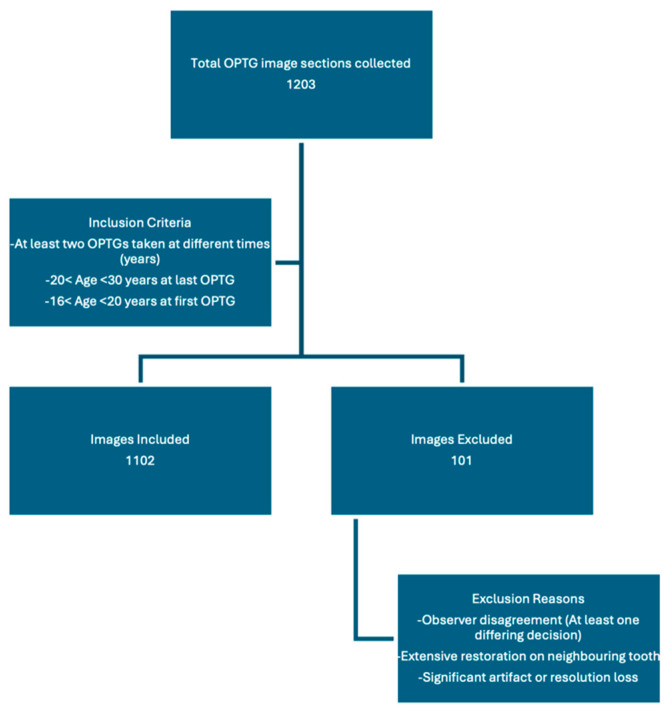
Flowchart of image selection process.

Labeling was performed using LabelIMG, an open-source software, in PascalVOC format. Each panoramic section was independently tagged by three blinded observers under a closed voting system. In instances where two observers concurred, that decision was accepted as the final label. Cases of complete disagreement were jointly reviewed, and images for which a consensus could not be achieved were excluded from further analysis, along with their corresponding earlier images from the same region. If a consensus was not reached for a specific right or left mandibular third molar in the most recent PAN, that region of the patient’s dataset was excluded from the analysis. Observers reached consensus in approximately 97% of cases, reflecting a high level of consistency among raters (estimated κ ≈ 0.8, indicating substantial agreement). In addition, images with large restorations covering the distal and/or occlusal surface of the adjacent second molar, significant artifacts, or resolution loss were excluded based on a consensus under the guidance of the oral and maxillofacial radiologist. As most patients had both right and left mandibular third molars in the dataset, the exclusion criteria were applied to specific tooth rather than the entire radiograph or patient. In total, 1203 PAN sections were collected and labeled from 296 unique patients. After excluding 101 sections for the reasons mentioned above, 1102 tagged images were included in the final study dataset (Figure 2). The distribution of the labels is shown in Table 1.

#### 2.2.2. Labeling Process

The labeling process followed a structured sequence: First, cropped sections were obtained from the most recent PAN images (final images) of all participants enrolled in the study. A fourth observer, independent of the clinicians responsible for labeling, anonymized and numbered all PAN images.

Labeling was performed as follows: If a participant had three images (A, B, and C), with C being the most recent, only image C was evaluated first. The eruption status of the tooth was classified as erupted, partially impacted, or impacted.

Operational definitions:

- Erupted: the occlusal surface of the third molar was at or above the occlusal plane of the adjacent second molar, with no overlying bone visible radiographically.
- Partially impacted: the occlusal surface was below the occlusal plane but partially exposed, with part of the crown still covered by alveolar bone or showing limited eruption space between the distal of the second molar and the anterior border of the ramus.
- Impacted: the third molar was entirely below the occlusal plane, with full bone coverage and/or evident spatial limitation or unfavorable angulation (e.g., mesioangular or horizontal position) in relation to the second molar.

**Figure 3:**
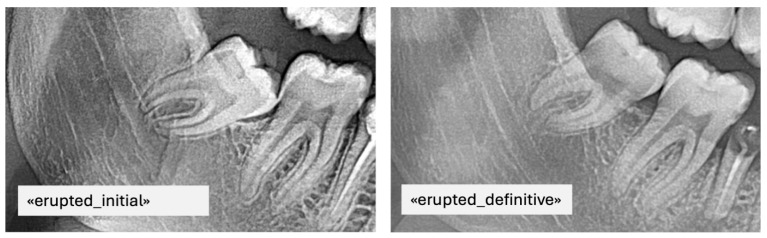
PAN images of the same patient with erupted mandibular third molars taken at different time points including the final label.

**Figure 4:**
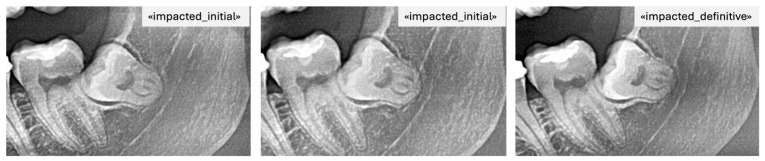
PAN images of the same patient with impacted mandibular third molars taken at different time points including the final label.

For instance, if it was labeled erupted, it was also labeled definitive as it was the last image (Figure 3). Consequently, images A and B were automatically tagged as erupted/initial, indicating that their future eruption status was uncertain at the time of acquisition (Figure 4).

The fourth observer uploaded all images into the LabelIMG (version 1.8.6; Tzutalin, GitHub) program and assigned the preliminary labels (for A and B) alongside the final labels within the software. The labels were then placed on the images by defining a region of interest (ROI) that included the mandibular third molars and surrounding structures according to the predefined boundaries. This completed the labeling process. An example of labeling performed in LabelIMG is shown in Figure 4. The collected label files were processed using a Python program that cropped the images based on the labeled regions (the dimensions of the bounding box were not standardized; they were drawn according to the area to be covered). In addition, within the same program, the regions were classified as left or right, and excess areas in the jaw region that did not affect the positional assessment were cropped.

#### 2.2.3. Model Training Process and Reference Model Selection

**Figure 5:**
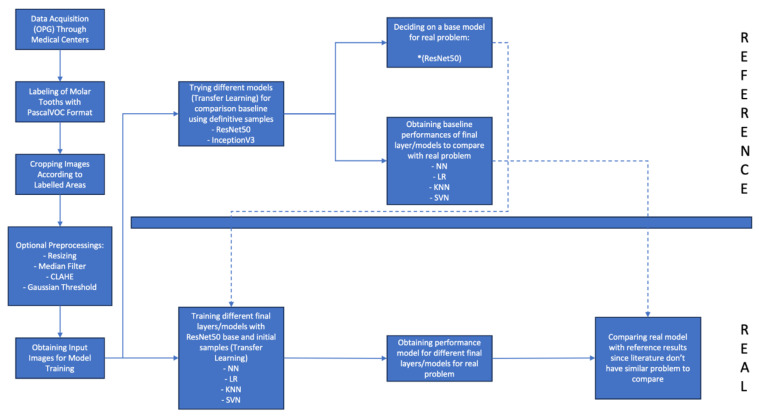
Flowchart of model training process.

A configurable neural network architecture and a reference model was developed for model training (Figure 5). The lack of studies in the literature required us to develop a reference model as well to compare training results of the model with a baseline. The structure provided flexibility in certain parameters, including the presence or absence of layers (e.g., optional rescale layer, dropout layer, fully connected layer) (Table 2). An overview to neural network architecture is provided below.

1. Data Augmentation Layer
2. InceptionV3 or ResNet50 Preprocessing Layer
3. Pretrained InceptionV3 or ResNet50
4. Rescaling Layer (Optional)
5. Global Average Pooling
6. Flatten Layer
7. Dropout Layer (Optional)
8. Fully Connected Layer (Optional)
9. Decision Layer (Softmax)

Once the model architecture was defined, training was performed using different parameter combinations, including learning rate, number of layers, and activation functions. The dataset was divided into 80% training and 20% test for both data groups (initial and definitive diagnoses). Within each training subset, 75% was used for training and 25% for validation. Detailed case counts for each class and data split are provided in Table 3.

Given that the dataset is not uniform, random oversampling is utilized to balance the data distribution for training datasets until all classes have same number of samples with most abundant class. Oversampling was applied exclusively to the training subsets to prevent data leakage into validation or test data. To ensure reproducibility, all random operations including data splitting and oversampling were conducted using a fixed random seed (sampler = RandomOverSampler(sampling_strategy = “not majority”, random_state = 1995)). The results obtained from different parameter settings were evaluated to assess model performance (Phase 1). Subsequently, based on the model performance, the parameter space was refined, and retraining was performed (Phase 2). Finally, in the optimized parameter space, preprocessing methods were cross-applied, and model performance was re-evaluated (Phase 3). The initial hyperparameter space for this study is shown below:

- Dropout Rate: 0, 0.15, 0.3
- Number of Nodes: 16, 32, 64, 128
- L2 Regulation: 0, 0.001, 0.01
- Learning Rate: 0.0005, 0.001, 0.005

In addition to conventional end-to-end CNN training, the convolutional neural networks were also used as feature extractors to evaluate the performance of classical machine learning models. After CNN training, activation values were extracted from the penultimate fully connected layer (before the Softmax decision layer), and these extracted features were then used to train logistic regression (LR), K-Nearest Neighbors (KNNs), and Support Vector Machine (SVM) classifiers. This approach allowed direct comparison between deep learning-based and traditional classifiers trained on CNN-derived features. The “Optimal Final Layer” rows in Table 4 and Table 5 reflect the best-performing classifier in each phase.

For reference model selection, the workflow was applied to both ResNet50, a 50-layer deep residual network known for its effectiveness in image classification, and InceptionV3, a deep learning architecture designed for image classification and object recognition that allows for the processing of more complex features with less computational power. Given that the dataset was not enough to train a ResNet50 or InceptionV3 from scratch, transfer learning is utilized with the pretrained version of mentioned models.

#### 2.2.4. Preprocessing Methods Used in This Study

**Figure 6:**
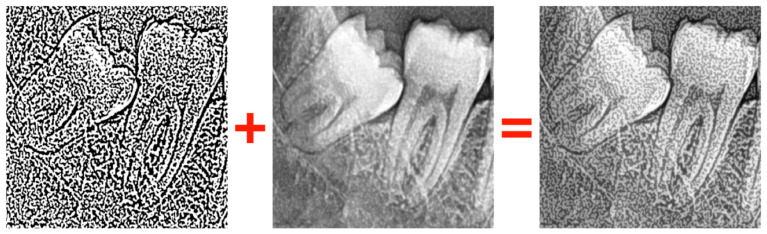
Merging the image processed with Gaussian thresholding with the original image.

Even though model architecture contains the model-specific preprocessing layer, additional preprocessing techniques are applied in this study:

- Resizer: A method used to adjust image dimensions to match the model input size.
- Median Filter: A filtering technique that reduces noise in the image, resulting in a smoother appearance.
- CLAHE (Contrast Limited Adaptive Histogram Equalization): A technique that enhances image contrast, making details more visible.
- Gaussian Thresholding: A segmentation method that classifies pixels based on whether they fall above or below a certain threshold (Figure 6).

The typical preprocessing parameters were as follows: 

CLAHE was applied with a clipLimit of 3.0 and a GridSize of (5, 5) to enhance local contrast. Median filtering was performed using a 3 × 3 kernel to suppress high-frequency noise. Gaussian thresholding was employed with 255 as maxValue, Gaussian_C as adaptiveMethod, Thresh_Binary as thresholdType, 11 as blockSize, and 2 as C to refine the segmentation. The image processed with Gaussian thresholding is merged with the original image while using an alpha value of 0.8 (Figure 6). Random horizontal flipping and random height/width shifts of ±20% were applied for data augmentation. All images were resized to 224 × 224 pixels (ResNet50) or 299 × 299 pixels (InceptionV3) before model training. The performance improvement observed in the Phase-3 ResNet50 model was primarily driven by the combined use of CLAHE and Gaussian thresholding, which enhanced edge contrast and overall feature representation.

During neural network training, a loss function is defined as *sparse_categorical_crossentropy* and Adam optimizer is utilized. In order to prevent overfitting, validation loss after each epoch is monitored and training was terminated if no improvement was seen in validation for the last 10 epochs. This method is known as early stopping. In order to improve optimization efficiency, a mini batch gradient descent is utilized, and the batch size is defined as 32. Model training also utilized data augmentation to improve model robustness and avoid overfitting. Used augmentation methods are random flip, random height, and random width.

In the study, the performance of classical machine learning algorithms was also evaluated. For this objective, following the culmination of the training process of the deep neural network, in addition to the calculation of performance metrics, the image features that were extracted by the neural network were obtained from the model that had undergone training. Specifically, activation values from the layer preceding the final decision layer (Softmax) were recorded as image features. The layer before the final decision layer is a fully connected hidden layer with an undefined number of nodes (which is defined during hyperparameter optimization) and a ReLu activation function. Output from the ReLu activation function were extracted and then utilized to train three classical machine learning models. In this approach, the trained neural network is used as a feature extractor for classical ML models, which is a well-known approach.

The machine learning models applied in this study were as follows:

Support Vector Machines (SVMs): An algorithm that defines decision boundaries between two classes. In this study, a one-versus-rest methodology is used for multi class classification with SVM.

K-Nearest Neighbors (KNNs): A classification algorithm that assigns labels based on the proximity of a data point to its neighbors.

Logistic Regression (LR): A statistical method that predicts the probability of an event occurring.

## 3. Results

### 3.1. Comparison of Model Performances

In the study, baseline models were developed using two different neural network architectures, InceptionV3 and ResNet50, based on definitive diagnosis images (the latest panoramic radiograph obtained from each patient). Performance comparisons between these models were conducted based on the F1 score (Table 4 and Table 5).

The InceptionV3 model exhibited consistent performance in definitive diagnoses; however, preprocessing methods had a detrimental effect on its performance (see Table 4). Conversely, the ResNet50 model experienced a notable enhancement in performance when preprocessing techniques were employed, achieving its maximum performance when cross-applied preprocessing methods were utilized.

Furthermore, the ResNet50 model, when complemented with classical machine learning methods (e.g., KNN and logistic regression), demonstrated superior performance in comparison to the deep learning-based final layers (Table 5).

### 3.2. The Effect of Hyperparameter Optimization

It has been found that hyperparameter optimization improves performance metrics such as precision, accuracy, and recall of the models (from Phase 1 to Phase 2). The ResNet50 model stood out as the reference model with the highest F1 score (0.829) on clear diagnoses (Table 5).

### 3.3. Performance on Initial Diagnoses

When the model trained on clear diagnoses (the final eruption status of the teeth) was retrained on initial diagnoses (earlier images with ambiguity regarding eruption status), a significant decline in model performance was observed (Table 6). This decrease was found to be highly likely related to the relatively small number of images used for training the model.

### 3.4. Overall Evaluation of the Results

From a clinical perspective, the model demonstrated a high level of accuracy in predicting whether a tooth would erupt or remain impacted, with accuracy rates of 83% and 75%, respectively (see Table 7). However, in the classification of partially impacted teeth, the model frequently misclassified them as fully impacted, achieving only 55% accuracy. This finding suggests that the model has limited discriminative ability in transitional cases, where the boundary between different eruption statuses is less distinct.

ROC-AUC analysis revealed that the model could accurately distinguish between fully erupted and fully impacted teeth; however, a decrease in classification performance was observed for partially impacted teeth (see Table 8 and Table 9).

**Figure 7:**
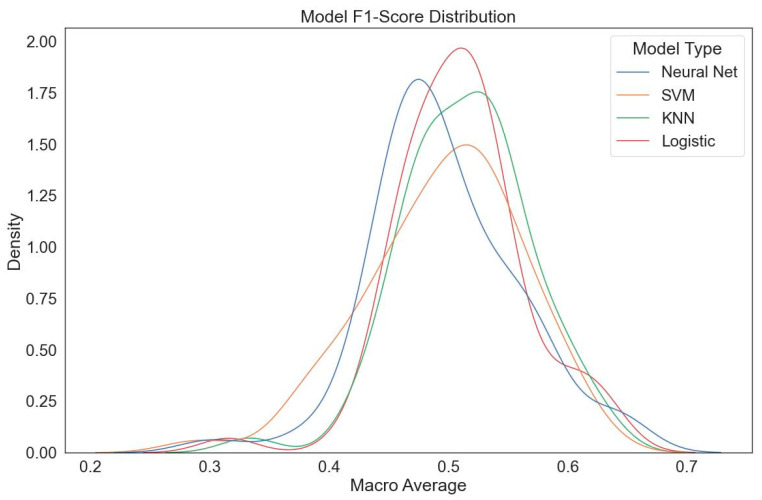
ResNet50 model F1 score distribution (optimized parameter space). The macro-average F1 distributions indicate that classical classifier heads (LR, KNN) outperform neural network heads in certain preprocessing configurations.

Nevertheless, upon examining the ROC-AUC values, it was concluded that the model provides a foundation that can be further optimized for future applications. In summary, the ResNet50 model used in this study demonstrated promising performance in predicting tooth eruption status within the field of oral and dental health. However, the decline in performance for initial diagnoses (during the dynamic intermediate eruption phase) and classification errors in the transition region indicate that the model requires enhancement through larger datasets and advanced optimization techniques (Figure 7).

## 4. Discussion

The utilization of radiological methodologies for the prediction of impacted third molar complications, and the objective support of prophylactic treatment decisions, is of paramount importance. Nevertheless, it is evident that the determination of the active eruption potential of teeth through radiological evaluation necessitates a more intricate analysis. As evidenced in this study, artificial intelligence (AI)-supported machine and deep learning methods—an increasingly prevalent approach in radiological diagnoses—offer a promising avenue for the standardization of this process and the guidance of clinical decision-making [14].

The capacity of artificial intelligence (AI) to enhance diagnostic precision is analogous to the efficacy of deep learning methodologies in the domain of early cancer detection. This enhancement is attained by AI’s capacity to discern radiographic alterations that are imperceptible to the human eye. In this context, it seems undeniable that AI-supported models possess the potential to detect microscopic changes in the soft and hard tissues surrounding teeth during the active eruption phase [15]. As a result, research into the capacity of AI to discriminate and categorize tooth positions has the potential to offer a perspective that extends beyond the scope of traditional dentistry methods. In addition, the use of AI to facilitate decision-making processes in the management of wisdom teeth demonstrates considerable potential for the early detection of caries or pathologies, including pericoronitis, in conjunction with the determination of tooth position. Nevertheless, the generalizability and clinical validation of such applications necessitate the availability of substantial and diverse datasets [16].

### 4.1. Interpretation of Findings

The present study demonstrates that the eruption potential of lower third molars can be predicted from panoramic radiographs using deep learning-based artificial intelligence models (ResNet50 and InceptionV3). The accuracy and F1 scores exceeding 80% achieved by the reference model are consistent with those reported in other studies in the literature [17,18]. However, when previous images were employed, a marked decrease in performance (F1 score of approximately 70%) was observed. From a literature-based perspective, this decline can be attributed to data imbalance and insufficient data from previous images [18]. ROC-AUC analyses revealed that the model provided high accuracy in discriminating relatively clear positions, such as fully erupted and fully impacted teeth. However, the classification performance decreased for partially erupted teeth (eruption period). This finding can be considered as an innovative contribution, given that previous studies have mostly focused on the evaluation of the positions or anatomical relationships of third molars [10,16].

### 4.2. Strengths of the Model and Comparison with Classical Machine Learning Methods

The study aimed not only to classify fixed features, but also to predict changes in the position of the lower third molars over time. In particular, the ResNet50 model stood out for its high accuracy (89%) and F1 score (83%) for unambiguous diagnoses, improving its performance through hyperparameter optimization and preprocessing methods such as CLAHE and Gaussian thresholding. However, in evaluations focused on ambiguous diagnoses (previous images), classical machine learning methods (e.g., logistic regression and KNN) showed superior performance compared to deep learning models [19,20,21]. A possible explanation for this phenomenon lies in the increased data requirements of deep learning models due to their complex architectures, which hinders their ability to generalize effectively with limited data, especially in future predictions. Conversely, classical machine learning methods showed an ability to produce more balanced results from the available data, due to their less complex structures. These methods also allowed for more effective decision support in initial diagnoses [22,23,24]. This emphasizes the necessity of customizing the model selection in accordance with the target problem and data structure.

In the future, training deep learning models with larger and more diversified datasets has the potential to substantially enhance performance, particularly in tasks that seek to make forward-looking predictions by monitoring the time-dependent changes in a tissue, as in our study. Concurrently, the utilization of classical machine learning methods as complementary tools in this process can contribute to a more balanced decision-making process. This approach clearly demonstrates the importance of methodological flexibility in medical AI applications.

### 4.3. Clinical Relevance

This study indicates that AI models have the potential to significantly contribute to clinical decision-making processes. Specifically, the ability to predict the eruption potential of impacted teeth may facilitate more objective decisions regarding extraction. The prediction of the future position of these teeth on panoramic radiographs can serve as a valuable tool in preventing position-related complications via prophylactic removal, such as pericoronitis or caries due to a tooth that will remain partially impacted. In contrast, maintaining impacted teeth in a healthy position with symptomatic treatments can reduce the need for unnecessary surgical procedures, their related complications, and financial expenses [4].

Furthermore, in clinical practice, the proposed model could assist clinicians in decision-making regarding the management of impacted teeth. When the model predicts a high likelihood of full eruption (accuracy ≈ 83%) or complete impaction (≈ 75%), the output can be used to support confidence in routine monitoring or early surgical planning. However, for partially impacted teeth, where accuracy is lower (≈ 55%), additional diagnostic imaging such as CBCT or referral for specialist consultation is advisable. Integrating this model as a supportive radiographic assessment tool may enhance early detection and individualized treatment planning.

### 4.4. Methodological Differences

The use of AI in medical imaging offers significant potential for studies focusing on diagnostic accuracy. However, significant gaps in adherence to methodological standards have been identified in the existing literature [25]. Some systematic reviews have suggested that AI models achieve high diagnostic accuracy in areas such as ophthalmology, breast pathology, and respiratory disease, but have highlighted the risk of inflated results due to methodological heterogeneity [23,26].

The perception of AI-based models as “black boxes” is a factor limiting their reliability in clinical applications. The decision-making processes of deep learning algorithms are generally opaque, and small errors that are barely perceptible to human observers, and therefore may be missed by quality control, carry the risk of the model failing altogether (adversarial attack). Therefore, open algorithms and standardized terminology are essential for wider and more reliable use in clinical settings [15,26]. In this context, the existing literature was thoroughly reviewed, and open-source software solutions were used to select the terminology for model training in the present study.

There are also significant differences in the way AI-related research is conducted and reported. In particular, there is a need for a universal, standardized format for reporting metrics, statistics, and clinical applicability. In recent years, guidelines such as CONSORT-AI, TRIPOD, and STARD-AI have been developed to address these issues. These are generally extended versions of existing guidelines, adapted specifically for AI-related trials [13,25]. In the present study, the TRIPOD guideline was preferred to ensure transparent and consistent reporting of AI research. As the lack of diagnostic accuracy and methodological standards in AI research is often highlighted, in line with the retrospective nature and clinical validity objectives of our study, the consolidated reporting standards offered by TRIPOD allowed for a systematic transfer of methodological processes. The use of this guideline has both increased the comparability of the study with other AI studies in the literature and strengthened its potential for integration into potential clinical applications.

Research conducted on the diagnostic accuracy of panoramic radiographs has indicated that PANs provide a lower level of diagnostic accuracy in comparison with stereoscanography and CBCT [7]. This emphasized the need to evaluate the performance of the AI models in this research, considering the limitations of panoramic radiographs. In accordance with the latest literature, the findings of this study explicitly suggest that to achieve better outcomes with artificial intelligence in dentistry, research must rely on robust datasets and maintain meticulous adherence to methodological standards.

In a study based on MobileNet V2—a lightweight, high-speed deep learning model developed by Google and optimized for mobile devices and embedded systems—an accuracy of 87% and an ROC-AUC of 0.90 were achieved in classifying caries in third molars. Although the study demonstrated high performance using a limited dataset (400 training and 100 test images), it relied on images from a single time point only, so the effect of temporal changes could not be assessed [27]. Another study investigating mandibular nerve and third molar segmentation used a single deep learning approach, such as a U-net model, on panoramic radiographs, achieving high accuracy rates and demonstrating success in identifying static anatomical structures. However, this study also presented evaluations based on a single learning model and a relatively limited dataset [26]. In the present study, the primary objective was to predict the position and eruption status of third molars over time. Using a larger dataset (1102 images) and two different models (InceptionV3 and ResNet50), ResNet50 emerged as the leading performer, achieving 89% accuracy and an F1 score of 83% for well-defined diagnoses. However, its accuracy dropped to around 70% when trained on previous images, suggesting that its ability to predict eruption status was lower than its ability to categorize current images. Given the critical role of dataset size in deep learning, these results have been attributed in the present study to data insufficiency and variations in image quality [23].

On the other hand, in the present study, classical machine learning methods such as SVM, KNN, and logistic regression were applied during the validation and testing process, and it was observed that the relatively low performance in predicting ‘”initial” diagnoses—attributed to a limited dataset—was improved compared to relying solely on deep learning models. In conclusion, while AI algorithms can deliver higher accuracy for more stable and well-defined challenges—such as caries classification—further optimization is required to analyze the dynamic processes that ultimately determine the final position of a tooth. Nevertheless, all of the above studies provide an important foundation for integrating AI into clinical decision-making in dentistry. On the other hand, in future studies, especially when working with a limited dataset and aiming for a classification-based result, focusing on traditional machine learning methods or integrating them into deep learning models seems to be a reasonable approach.

In a comparable study, the stages of third molars were determined through labeling based on the consensus of two observers, while a third observer served as an arbiter in cases of disagreement. Inter-observer consistency was measured using the Rank-N recognition rate (Rank-N RR), and algorithm performance was evaluated according to its alignment with the observers’ decisions [14]. In contrast, three independent observers were employed in the present investigation, and only labels on which all observers unanimously agreed were included. Both approaches are believed to strengthen inter-observer reliability and increase dataset validity.

Additionally, in the study by Tobel [28], the region of interest surrounding the lower third molars was determined manually with bounding boxes in a manner similar to ours; however, the AlexNet model was retrained via transfer learning to classify third molar stages. In comparison, the CNN models utilized here, particularly ResNet50, feature a deeper and more modern architecture, making them appear more suitable for predicting dynamic processes rather than addressing static tasks such as age estimation, as pursued by Tobel et al. These observations indicate that both the labeling strategy and neural network architecture significantly influence the reliability and accuracy of dynamic and static assessments in radiographic data, providing valuable insights for the design and refinement of future research methodologies.

### 4.5. Limitations

This study has several limitations. Firstly, the dataset used was collected from a single center and therefore offers a limited range of numerical data. Although the study examined a dynamic event—the active eruption phase of lower third molars—that occurs within a specific time frame, this may limit the generalizability of the model and its performance in different scenarios [28]. In addition, as the final positions of the third molars were determined based solely on the radiographic assessments of three clinicians and not verified by clinical examination or CBCT, the risk of error, although minimal, cannot be completely ruled out [13,26]. It should also be noted that the boundary definition of partially impacted teeth is inherently ambiguous on panoramic radiographs as transitional eruption stages cannot always be clearly differentiated radiographically. Therefore, orthogonal or clinical information, such as soft tissue evaluation, may be required to accurately characterize these cases. As an unvalidated clinical diagnosis may compromise the certainty of the model’s diagnostic accuracy, it is recommended that similar models developed in future studies be trained using diagnoses that have been clinically validated or verified by advanced imaging techniques. In addition, the potential benefits and limitations of the model in clinical applications—particularly in decision support—warrant further discussion. While the model may assist clinicians in the early diagnosis of impacted teeth, further refinement and retesting are required for different demographic groups (e.g., older individuals) and different scenarios (e.g., caries or the onset of adjacent pathologies).

During the labeling process, classification challenges were encountered in the transitional regions, which adversely affected the model’s performance in classifying partially impacted teeth. Consequently, the use of supplementary evaluation criteria, such as the Pederson scale, may prove beneficial for more accurately delineating samples in the eruption phase [15]. Furthermore, a potential limitation of this study is that the dataset split was performed at the image level rather than the patient level, which may have introduced a degree of data leakage across subsets and led to a slightly optimistic estimate of model performance. Moreover, because multiple sections and time points from the same patients were included, complete statistical independence between samples could not be ensured, which may have introduced intra-patient correlation. As the study primarily aimed to evaluate model performance at the initial diagnostic stage, confusion analysis was limited to the initial dataset, while the definitive dataset served as the reference baseline.

Nevertheless, it should be noted that the evaluation of “eruption potential” in this study was based solely on positional data. The integration of additional anatomical parameters, such as skeletal structure and bone density, as well as genetic and anthropological factors, and the training of AI models on more comprehensive datasets, may serve as a guiding framework for future long-term research.

This research should be regarded as a pilot study that has established a foundational framework. Enhanced model performance may be achieved by employing larger, multi-center datasets. Future studies using larger, multi-center datasets and alternative balancing approaches, such as class weighting or SMOTE, are warranted to validate these findings. Furthermore, the development of additional labeling categories could aim to improve classification accuracy in transitional regions, specifically during the eruption phase when teeth are partially impacted [4]. AI models focus on establishing the connections between inputs and the desired outputs. Therefore, it is essential that these models concentrate on relevant factors and avoid being misled by erroneous data. Operator dependent repeatability and thereby sensitivity of diagnosis need to be further investigated for validity of the system.

### 4.6. Suggested Directions for Future Research

In order to enhance the clinical applicability of integrating AI models into radiological diagnostic and prognostic methods, it is recommended that future studies focus on the following aspects:

- The utilization of high-resolution images (e.g., the employment of CBCT instead of conventional radiographs)
- The incorporation of standardized yet diverse data inputs
- The engagement of multiple observers while minimizing inter-observer variability
- The integration of practical technologies such as mobile devices and cloud computing. The conducting of tests across different demographic groups and scenarios
- In order to achieve generalizability, it is essential to consider factors such as dataset size and the expected performance metrics (e.g., detection, classification, prediction, treatment recommendations) when selecting the appropriate model.
- Furthermore, undertaking prospective studies for clinical validation is recommended.

These strategies can help optimize service processes and improve the integration of AI in clinical practice [7,13].

The present study demonstrated that AI-supported classical and deep learning models have the potential to serve as a predictive tool in determining the eruption status of third molars. Despite its limitations, the model’s performance is promising in terms of its applicability in clinical decision-making processes. These findings represent an important step toward understanding how artificial intelligence can be employed to support dental diagnostic procedures.

## 5. Conclusions

This study demonstrated that deep learning models, particularly ResNet50 and InceptionV3, can effectively predict the eruption status of mandibular third molars using panoramic radiographs. These models showed high accuracy for clearly impacted or erupted teeth, while performance was lower in partially impacted cases.

Overall, the findings highlight the potential of AI-based tools to support clinical decision-making in third molar management. With access to larger datasets, improved optimization methods, and enhanced imaging inputs, such models could evolve into reliable, routine decision support systems in dental practice.

## Figures and Tables

**Table 1:** Class distribution of labeled images.

Label Definition	Initial (n.268)			Definitive (n.834)		
	Impacted	Partially Impacted	Erupted	Impacted	Partially Impacted	Erupted
Label number	118	89	61	147	119	568

**Table 2:** Hyperparameters modifying the neural network architecture.

Parameter Definition	The Effect of the Parameter on the Model
Dropout Rate	It is the rate used in the dropout layer within the model architecture. As the rate increases, it is expected to prevent overfitting. When this value is set to “0”, the dropout layer is removed from the model architecture.
Number of Nodes	It is the number of nodes to be used in the fully connected layer within the model architecture. When this value is set to “0”, the fully connected layer is removed from the model architecture.
L2 Regularization *	This value is used to enhance the effect of L1 and L2 regularization applied in the fully connected and decision layers. Unless otherwise specified, the default values for L1 and L2 regularization are set to “0”.
Learning Rate	The learning rate is a parameter that determines the learning speed of the optimization method chosen for model training.

* L1 and L2 regularization are techniques used to prevent overfitting in neural networks. L1 regularization (Lasso) encourages sparsity by reducing some weights to zero, while L2 regularization (Ridge) discourages large weight values, promoting a more balanced distribution.

**Table 3:** Distribution of cases in initial and definitive diagnosis datasets (before oversampling).

Cohort	Class	Train	Validation	Test
Initial	Impacted	70	24	24
	Partially Impacted	53	18	18
	Erupted	18	6	12
Definitive	Impacted	340	144	114
	Partially Impacted	88	30	29
	Erupted	71	24	24

Note: Validation counts were derived from the training subset using a 75/25 split (i.e., validation ≈ 33% of training samples).

**Table 4:** Optimal model performances obtained in the phases of the InceptionV3 reference model study (definitive diagnosis). Results are based on the test set (*n* = 167; impacted = 114, partially impacted = 29, erupted = 24).

Model Performance Metrics	Phase 1	Phase 2	Phase 3
Accuracy	0.850	0.856	0.850
Recall	0.816	0.821	0.804
Precision	0.794	0.795	0.788
F1 Score	0.800	0.803	0.792
Optimal Final Layer	Logistic Regression	Logistic Regression	Logistic Regression

**Table 5:** Optimal Model Performances Obtained in the Phases of the ResNet50 Reference Model Study (Definitive Diagnosis). Results are based on the test set (*n* = 167; impacted = 114, partially impacted = 29, erupted = 24).

Model Performance Metrics	Phase 1	Phase 2	Phase 3
Accuracy	0.892	0.880	0.892
Recall	0.784	0.789	0.814
Precision	0.843	0.833	0.846
F1 Score	0.808	0.809	0.829
Optimal Final Layer	Logistic Regression	Neural Network	KNN

**Table 6:** Optimal model performances obtained in the phases of the ResNet50 reference model study (initial diagnosis). Results are based on the test set (*n* = 54; impacted = 24, partially impacted = 18, erupted = 12).

Model Performance Metrics	Phase 1	Phase 2	Phase 3
Accuracy	0.629	0.648	0.704
Recall	0.620	0.634	0.713
Precision	0.683	0.672	0.707
F1 Score	0.639	0.647	0.705
Optimal Final Layer	KNN	Neural Network	KNN

**Table 7:** Confusion matrix of the optimal model predictions (true class vs. predicted class).

True Class (Definitive)	Predicted Impacted (from Initial Image)	Predicted Partially Impacted (from Initial Image)	Predicted Erupted (from Initial Image)	Support
Impacted	18 (75%)	3 (12.50%)	3 (12.50%)	24
Partially Impacted	7 (38.88%)	10 (55.55%)	1 (5.55%)	18
Erupted	1 (8.33%)	1 (8.33%)	10 (83.33%)	12

**Table 8:** Optimal model ROC-AUC values obtained in the phases of the InceptionV3 reference model study.

	Phase 1	Phase 2	Phase 3
Impacted Definitive	0.928	0.939	0.944
Partially Impacted Definitive	0.885	0.881	0.872
Erupted Definitive	0.930	0.932	0.929

**Table 9:** Optimal model ROC-AUC values obtained in the phases of the ResNet50 reference model study.

	Phase 1	Phase 2	Phase 3
Impacted Definitive	0.947	0.970	0.926
Partially Impacted Definitive	0.826	0.873	0.794
Erupted Definitive	0.929	0.948	0.901

## Data Availability

Data available on request due to restrictions eg privacy or ethical.
